# Factors That Predict the Growth of Residual Nonfunctional Pituitary Adenomas: Correlations between Relapse and Cell Cycle Markers

**DOI:** 10.1155/2018/1876290

**Published:** 2018-07-10

**Authors:** Petr Matoušek, Petr Buzrla, Štefan Reguli, Jan Krajča, Jana Dvořáčková, Radim Lipina

**Affiliations:** ^1^Department of Otorhinolaryngology and Head and Neck Surgery, University Hospital Ostrava, Czech Republic; ^2^Department of Pathology, University Hospital Ostrava, Czech Republic; ^3^Department of Neurosurgery, University Hospital Ostrava, Czech Republic; ^4^Department of Radiology, University Hospital Ostrava, Czech Republic

## Abstract

**Introduction:**

Nonfunctional pituitary adenomas are treated surgically, and even partial resection can improve or eliminate clinical symptoms. Notably, progression requires further intervention, which presents an increased risk, especially in older patients. This study investigated whether the histopathological characteristics of nonfunctional adenomas could predict recurrence.

**Materials and Methods:**

Data were obtained retrospectively from 30 patients who underwent surgery for the partial resection of pituitary adenomas. Remnant tumor growth was observed in 17 patients, while the residual tumor was unchanged more than 7 years after surgery in 13 patients. Statistical analysis was performed to investigate correlations between remnant tumor progression and tumor histopathological findings, including protein expression of p21, p27, p53, and Ki-67.

**Results and Discussion:**

Remnant tumors that demonstrated regrowth showed significantly higher protein expression of p21 and Ki-67. Expression of the p53 tumor suppressor was also higher in this group, but the difference was at the limit of statistical significance.

**Conclusion:**

Tumors with high expression of p21 and p53 and with a high Ki-67 index were more likely to show residual pituitary adenoma progression. Such cases should undergo frequent radiological examination and timely reoperation, and radiosurgery should be considered.

## 1. Introduction

Pituitary adenomas are generally considered benign tumors. They have a positive prognosis following surgical removal, and residual tumors tend to have a low growth rate. Pituitary adenomas are frequently diagnosed as the result of incidental findings, and as is generally the case with incidentaloma, they are often followed up for years without any progression in size or any development of clinical problems.

In patients with functional adenomas, total resection is needed to fully treat the clinical issues. In contrast, in patients with nonfunctional adenomas, postoperative improvement in chiasmatic syndrome can be achieved by simple decompression of the optic chiasm. However, both our experience and published data show that recurrence occurs more frequently than is generally expected following both partial and total resection; recurrence is particularly apparent in cases with partial resection, which shows recurrence rates as high as 64% over 5 years [1].

In general, the recurrence rate of neoplasm depends not only on the radicality of resection but also on proliferative activity of tumor. This activity may be evaluated using p21, p27, Ki-67, and p53 as cell cycle and proliferation markers. Notably, p21 and p27 are considered as cell cycle markers and function as cyclin-dependent kinase (CDK) inhibitors that belong to the CIP/KIP family [2, 3]. Ki-67 is a reliable cell proliferation marker that is used widely in biomedical studies, and the p53 oncoprotein plays major roles in apoptosis, DNA repair, genomic stability, and angiogenesis inhibition, while mutated p53 supports tumorigenesis [4].

We hypothesized that there is a correlation between above-mentioned cell cycle proliferation markers and residual adenoma growth. The aim of this study was to identify the histopathological features of adenomas that are associated with tumor growth by retrospectively analyzing a patient cohort with postoperative residual pituitary adenoma.

## 2. Materials and Methods

The study cohort comprised 32 patients who were admitted to the Department of Neurosurgery of University Hospital Ostrava between 1999 and 2012 for the partial resection of nonfunctional pituitary adenomas. Partial resection (as opposed to total resection) had been performed in these patients for one of two reasons. First, the consistency or location of some adenomas (e.g., in the cavernous sinus) precluded total resection, so the main goal was to decompress the optic chiasm. Alternatively, the tumor remnant was not related to the strategy of the initial surgery. Rather, in these cases, the residual adenoma was discovered during routine magnetic resonance imaging (MRI) scans 3 months after operation and was not an indication for reoperation, since clinical signs had regressed during this period.

We obtained paraffin blocks of tissue and specimens for all 32 patients from the archive of the Pathology Institute at University Hospital Ostrava. Following histological and immunohistochemical analysis, two patients were excluded from the study cohort due to low sample quality. The adenomas of the remaining 30 patients comprised the definitive study group and were clinically evaluated to be nonfunctional adenomas.

The patients primarily underwent microscopic or endoscopic transcranial or transsphenoidal surgery. Recurrence was treated by microscopic or endoscopic transsphenoidal surgery and/or with radiosurgery. The following clinical and histopathological parameters were analyzed:

1. Patient sex and age at the time of the primary operation
2. The size of the residual adenoma (unidimensional size, i.e., longest tumor diameter in the transverse, coronal, or sagittal planes) as established by MRI 3 months after operation
3. Duration of postoperational monitoring of the residual adenoma and the time to radiologically observed progression requiring reoperation and/or radiosurgery
4. Histopathological determination of the Ki-67 proliferation index and expression levels of the p53 tumor suppressor factor and the p21 and p27 cell cycle markers

Samples taken at the time of the primary operation were analyzed histologically in both groups, that is, in patients with progressive versus nonprogressive residual pituitary adenoma. Samples from the paraffin blocks were subjected to hematoxylin and eosin staining and to immunohistochemical analysis using antibodies against the following: ACTH (polyclonal, 1:500, Cell Marque); FSH (polyclonal, 1:100, Cell Marque); GH (polyclonal, 1:500, Cell Marque); LH (polyclonal, 1:500, Cell Marque); prolactin (polyclonal, 1:500, Cell Marque); TSH (polyclonal, 1:500, Cell Marque); p21 (clone SX118, 1:50, Dako); p27 (clone SX53G8, 1:50, Dako); p53 (clone DO-7, 1:800, Novocastra); and Ki-67 (clone MIB-1, 1:150, Dako). Patient data were processed anonymously. This retrospective analysis was approved by the Ethics Committee of the University Hospital Ostrava.

## 3. Results

### 3.1. Characteristics of Patients with Progressive versus Nonprogressive Adenoma

We compared the characteristics of patients with progressive versus nonprogressive residual pituitary adenoma. All patients had previously undergone partial resection of a functional pituitary adenoma. The first group (progressive group) comprised 17 patients (8 women and 9 men; median age at surgery, 49 years) with progressive residual pituitary adenoma. The residual tumor size (largest diameter) ranged from 5 to 33 mm (median, 18 mm). Remnant tumor growth was observed from 1 to 6.1 years (median, 4 years) after the primary operation, and microscopic or endoscopic transsphenoidal surgery was subsequently performed. Additional radiosurgery was performed in 2 cases, in which the residual tumors persisted even after follow-up surgery.

The second group (nonprogressive/control group) comprised 13 patients (4 women and 9 men; median age at surgery, 57.5 years) with nonprogressive residual pituitary adenoma. The residual tumor size (largest diameter) ranged from 3 to 34 mm (median, 12.9 mm). The patients were monitored for at least 7.2 years after operation (median, 8.9 years) up to a maximum of 11.4 years.

**Figure 1:**
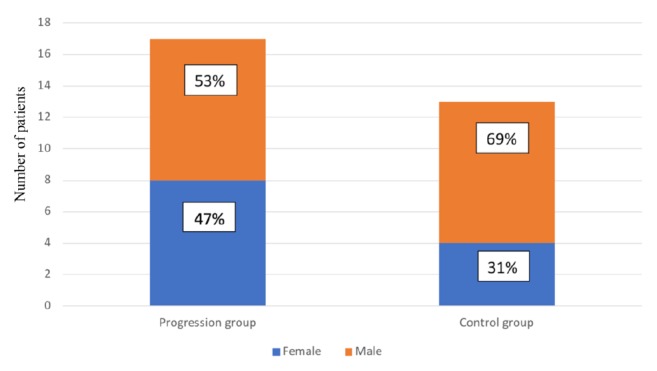
There was no significant difference in the male-to-female ratio in groups of patients with progressive versus nonprogressive adenoma (control) (*p *= 0.4651).

**Figure 2:**
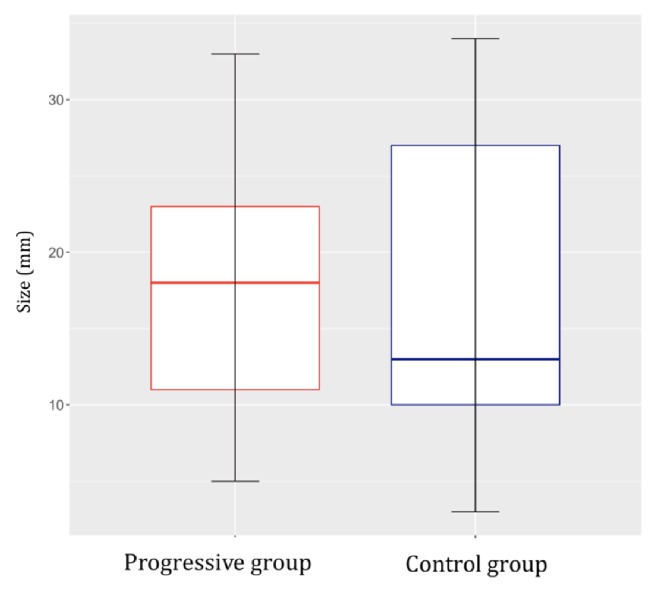
There was no significant difference in the postoperational size (largest diameter) of residual adenoma as determined by MRI in groups of patients with progressive versus nonprogressive adenoma (control) (*p *= 0.7693).

**Figure 3:**
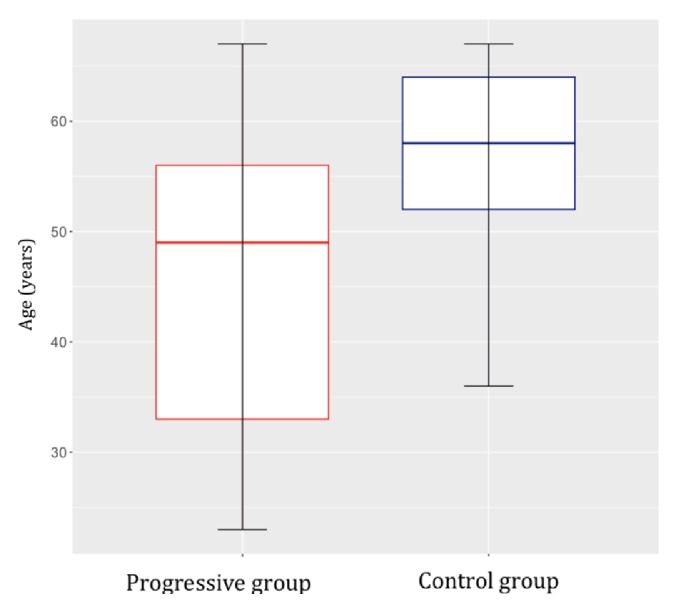
The median age of the group of patients with nonprogressive adenoma (control) was significantly higher at the time of the primary operation than the age of patients with progressive adenoma (*p *= 0.0381).

**Figure 4:**
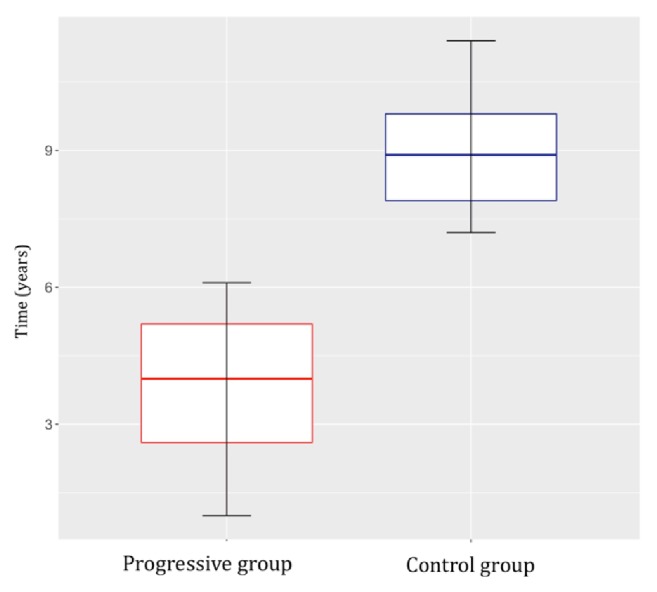
Significantly less time elapsed between the primary operation and adenoma progression compared to the length of the monitoring period of patients in the nonprogressive (control) group (*p*<0.001).

Fisher's exact test and the nonparametric unpaired Kruskal-Wallis test were used to compare the characteristics of the two study groups. There were no significant differences in sex or residual adenoma size (Figures 1 and 2). In the nonprogressive group, patients were older at the time of the primary operation (*p *= 0.0381, Figure 3). In the progressive group, significantly less time elapsed between the primary operation and progression compared to the monitoring time of patients in the nonprogressive group (*p* < 0.001, Figure 4).

### 3.2. Statistical Analysis

**Figure 5:**
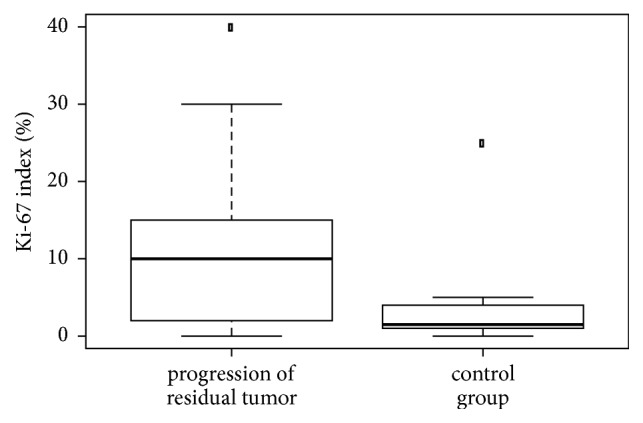
Comparison of the Ki-67 proliferation index in patients with progressive versus nonprogressive adenoma (control). The Ki-67 proliferation index was higher in the progressive group (*p *= 0.04).

**Figure 6:**
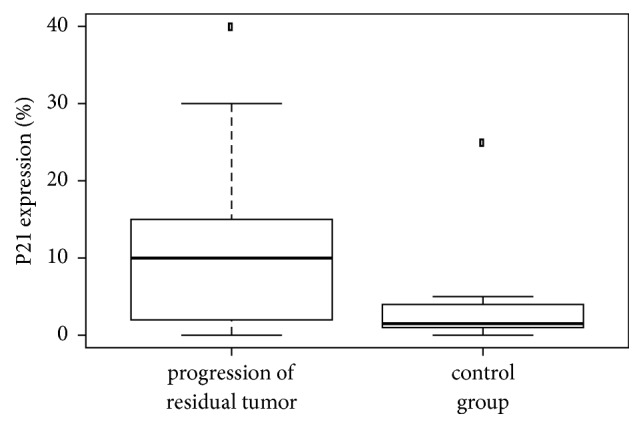
Comparison of the expression of p21 in patients with progressive versus nonprogressive adenoma (control). The p21 protein expression was higher in the progressive group (*p *= 0.04).

**Figure 7:**
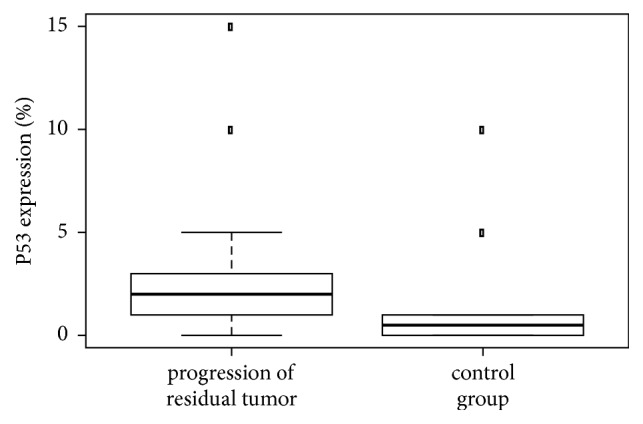
Comparison of protein expression of the p53 tumor suppressor in patients with progressive versus nonprogressive adenoma (control). The p53 protein expression was higher in the group with progression; however, the difference was not statistically significant (*p *= 0.0528), possibly due to the small group sizes.

The nonparametric unpaired Kruskal-Wallis test was used to evaluate differences between the two study groups (progressive versus nonprogressive residual pituitary adenoma) in p21, p27, and p53 expression and in the Ki-67 proliferative index. Keeping in mind the number of patients and variances in values, we investigated whether the Ki-67 proliferative index was significantly different in the two groups. Proliferative activity was significantly higher (*p*_(K-W)_ 0.04, Figure 5) in the group with progressive residual adenoma than in the group with nonprogressive residual adenoma, as was p21 expression (*p*_(K-W)_ 0.04, Figure 6). The median p53 expression level differed between the groups: expression was higher in the group with progressive residual adenoma, but this was not statistically significant (*p*_(K-W)_=0.0528, Figure 7). This may have been due to the low number of patients in the study. p27 expression was highly variable, and no conclusions could be drawn regarding its expression in the two groups.

### 3.3. Histopathological Characteristics of Adenomas from Patients with versus without Progressive Adenoma

**Figure 8:**
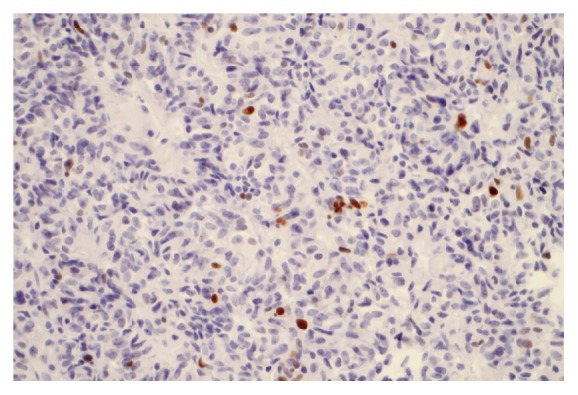
The p21 protein was expressed in up to 15% of tumor cells. Immunohistochemical staining was viewed at 40X magnification.

**Figure 9:**
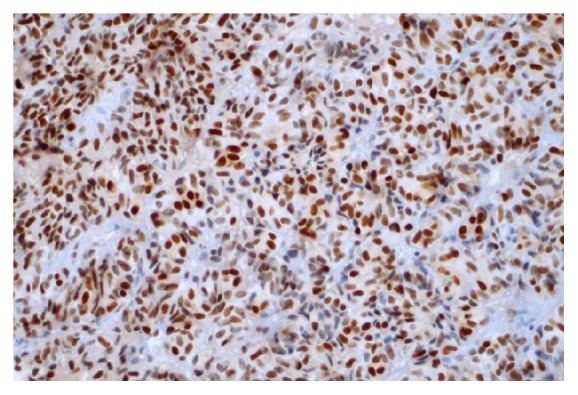
The p27 protein was expressed in approximately 90% of tumor cells. Immunohistochemical staining was viewed at 40X magnification.

**Figure 10:**
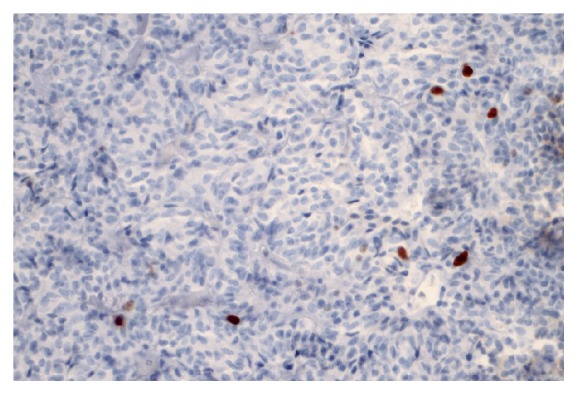
Proliferative activity (Ki-67 expression) was observed in up to 5% of tumor cells. Immunohistochemical staining was viewed at 40X magnification.

Next, we analyzed the immunohistochemical findings, the WHO classification, and the AFIP classification in the two groups of patients. In the progressive group, the adenomas were classified as gonadotrophic in 3 cases, as null cell adenoma in 5 cases, and as plurihormonal in 10 cases. Immunohistochemical analysis revealed moderate p21 protein expression in 0% to 40% of the tumor volume (Figure 8). Low p27 protein expression levels were found in all adenomas in 75% to 90% of the tumor volume (Figure 9). Low p53 protein expression was found in 0% to 15% of the tumor cells in all adenomas; the expression level was 10% or higher in 2 cases. Use of the anti-Ki-67 antibody showed that the proliferation activity in the adenomas ranged from 1% to 10% (Figure 10), with activity >3% in 15 of these cases.  Table 1 summarizes these results.

In the nonprogressive group, the adenomas were classified as plurihormonal in all 13 cases. Immunohistochemical analysis revealed low p21 protein expression in 0% to 25% of the tumor volume. p21 expression could not be determined in one case. Low p27 protein expression was found in all adenomas in 25% to 90% of the tumor volume. p27 protein expression could not be determined in two cases. Low p53 protein expression was found in 0% to 10% of tumor cells in all adenomas. One case showed expression in 10% of tumor cells, and p53 protein expression could not be determined in one case. Use of the anti-Ki-67 antibody showed that the proliferation activity in all adenomas ranged from 1% to 2%.  Table 2 summarizes these results.

## 4. Discussion

This study compared the histopathological characteristics of the adenomas in two groups of patients following partial resection of nonfunctional pituitary adenoma. In the first group, termed the progressive group, additional surgical intervention was necessary due to growth of the residual adenoma. In the second group, termed the nonprogressive group, long-term monitoring found no changes in the residual tumors. Other clinical data were not compared, such as clinical symptoms, invasive adenoma size before the primary operation, and comorbidities. There were no differences between the groups in terms of the residual adenoma size or sex distribution. There was a statistically significant difference in the median patient age at the time of the primary operation with that of the nonprogressive group being 8.5 years higher. There was also a significant difference in the monitoring time, which is important to consider when evaluating the results: in the progressive group, the median duration of monitoring before progression was 4 years, whereas in the nonprogressive group, the median monitoring time was 8.9 years. We expected the histopathological characteristics of the adenomas to differ between the groups, with higher expression of markers of growth in the progressive group.

Previous studies showed that recurrence or progression is relatively common in cases with residual adenoma, occurring in 7% to 24% of cases that undergo total resection of nonfunctional pituitary adenomas and in 47% to 64% of cases in which partial resection is performed [1, 5]. These percentages suggest that radicality of resection is the main factor that determines postoperational recurrence. Notably, the adenomas in our study cohort were partially resected either intentionally (when their characteristics or invasive nature precluded radical resection) or unintentionally (when residuum was discovered during routine MRI 3 months after operation). However, all of these patients experienced regression of clinical symptoms and thus were only monitored subsequently. It is now possible to use perioperative radiological methods such as ultrasound [6] or MRI [7] not only to determine radicality but also to continue the operation if residual tumor is found to be safely removed by surgery. Thus, radical resection is increasingly possible, which reduces the risk of recurrence [5, 7]. These methods can also help achieve radical resection in some cases in which partial resection was unintentional.

Regarding histopathological factors that might predict the progression of nonfunctioning residual adenoma, we focused on proliferative and cell cycle regulation markers that have already been discussed in the scientific literature. The p21 and p27 markers were used to evaluate the cell cycle in primarily resected adenomas. p21 is a 21 kD protein that belongs to the CIP/KIP (CDK-interacting protein/kinase inhibitory protein) family of cyclin-dependent kinase (CDK) inhibitors. Notably, p21 binds to and inhibits the activity of the cyclin B/CDK1, A/E/CDK2, and D/CDK4/6 complexes and thus regulates cell cycle progression in the G1 and G2 phases [2]. Reduced p21 expression or a lack of p21 is mainly observed in tumors with mutant p53 [8]. In our work, we found significantly higher p21 expression in the progressive group than in the nonprogressive group. However, published research has not yet confirmed that there is a relationship between p21 expression and the likelihood of progression. Lee et al. investigated whether cell-cycle factors could predict recurrence in functioning adenomas. While p16, the pRB protein, and cyclin D1 were associated with an increased risk of recurrence, p21 was not found to be a prognosis marker [9]. The role of p27 is similar to that of p21, although p27 binds only to the cyclin A/CDK2, E/CDK2, and D/CDK4 complexes [3]. According to the AFIP (Armed Forces Institute of Pathology) classification, the p27 gene is not mutated in most pituitary adenomas but shows reduced expression in corticotropic and recurring adenomas [8]. We found that p27 expression was highly asymmetric and therefore could not be evaluated.

The p53 tumor suppressor acts to prevent cancer and has roles in apoptosis, genomic stability, and inhibition of angiogenesis. It also activates DNA repair proteins and, together with p21, induces cell cycle arrest so that DNA errors can be repaired [4]. Another important role for p53 is to initiate apoptosis (i.e., programmed cell death) when there is irreversible DNA damage [10]. Accordingly, we investigated p53 expression in the adenomas of the 30 patients in this study. We found that p53 expression was higher in the progressive group. Interestingly, reports regarding the relationship of p53 and progression are contradictory: while two studies demonstrated that high p53 expression levels were a reliable indicator of tumor aggression and recurrence [9, 11], two other studies did not confirm this [12, 13].

The Ki-67 labelling index is generally considered a reliable marker of proliferation. Indeed, nuclear expression in more than 3% of tumor cells is an indicator of an aggressive adenoma that will have a greater tendency to recur [8, 14]. Our findings were consistent with this, with the progressive group having significantly higher Ki-67 expression. According to the WHO classification (2004), a Ki-67 proliferative index greater than 3%, along with diffuse and high p53 expression (i.e., p53 expression in more than 10% of tumor cells in the field of view at high magnification), can be regarded as a reliable indicator of aggressiveness and more frequent recurrence [14]. Adenomas of this type are classified as atypical, that is, as a transitional type that is between typical adenomas and pituitary carcinomas. Despite this, the prognostic value of the Ki-67 proliferative index has been questioned in the neurosurgical literature. Šteňo et al. [15] investigated whether the histopathological markers HMGA-1 Li and Ki-67 were associated with the recurrence/progression of residual pituitary adenoma. While they found no relationship regarding HMGA-1 Li, Ki-67 values over 2.2% were always associated with the growth of residual adenomas. As a result, the authors recommended more frequent MRI follow-ups in this group of patients. In contrast, Česák et al. [16] could not confirm a correlation between the Ki-67 proliferative index and recurrence/progression; rather, they found that the residual growth rate was inversely proportional to the age of the patient. This correlation was also observed in our study cohort, with patients in the progressive group being significantly younger (median age) than those in the nonprogressive group. In accordance with our findings, Losa et al. [17] described residual recurrence/growth in 19% of patients with nonfunctional adenomas, with risk factors that included younger age at the time of the radical primary operation. Those authors discuss postoperative radiotherapy as a way to prevent residual adenoma progression.

After evaluating our results and the literature, it seems that it will be beneficial to use multiple criteria, including clinical and radiological data, to supplement histopathological data in determining the prognosis of residual pituitary adenomas. Two recent studies have introduced new clinicopathological classifications for comprehensive prognostic grading of pituitary adenomas. The first study, by Trouillas et al. [18], used MRI to diagnose cavernous or sphenoid sinus invasion and categorized tumors according to size (<10 mm, ≥10 mm, and >40 mm), hormone production, grade (grade 1a, noninvasive; 1b, noninvasive and proliferative; 2a, invasive; 2b, invasive and proliferative; and 3, metastatic), and detection of at least 2 of 3 proliferation markers (>2 mitoses at 10 HPF (high-power field), Ki-67 proliferative index >3%, and high p53 expression in >10 nuclei at 10 HPF). The authors concluded that grade 2b tumors have an increased possibility of recurrence.

The second study, published by Saeger et al. in 2016 [19], followed the classification of the first study but altered the invasion assessment so that invasion is only radiologically proven by MRI and supplements the number of invasion sites (into the cavernous sinus, the sphenoid sinus, and the clivus). This classification categorized adenomas into two groups (grade 1, nonproliferative adenoma; grade 2, proliferative adenoma) with descriptions of invasion (grade 1a, 2a, no invasion; grade 1b, 2b, invasion at one location; grade 1c, 2c, invasion at two or more locations; and grade 2d, carcinoma). To prove the invasion of atypical adenomas, which are associated with a higher probability of recurrence and clinically aggressive progression, they proposed using the presence of at least 2 of 3 proliferation markers.

## 5. Conclusions

Multiple factors can influence the growth of residual pituitary adenomas, including patient age, tumor histopathology (particularly cell cycle and proliferation markers) and, most likely, adenoma growth characteristics and consistency. Based on our results, we conclude that the growth of residual adenomas is more likely in adenomas that have higher expression levels of the p21 cell cycle marker and the p53 tumor suppressor marker as well as a higher Ki-67 proliferative index. In patients with these findings, early surgical resection of the remnant tumor and/or radiosurgery should be considered, especially in younger patients.

## Figures and Tables

**Table 1:** Overview of the clinical and histopathological characteristics of patients with adenoma progression. *∗* indicates residual adenoma size as measured at the largest diameter.

Patient	Sex	Age	Residual adenoma size, mm*∗*	Time to residual adenoma progression, years	p21 expression	p27 expression	p53 expression	Ki-67 expression
1	F	63	13	3.6	10	75	5	5
2	F	55	23	6.1	5	90	2	5
3	M	67	29	5.5	15	90	3	10
4	M	33	33	4.2	40	90	2	5
5	M	61	28	1	30	90	15	10
6	M	54	12	5.9	10	90	2	3
7	F	29	11	5.2	2	75	2	4
8	F	45	22	1.5	5	90	0	2
9	M	44	6	4	15	75	1	3
10	F	56	15	1.5	30	90	10	8
11	M	29	18	6	0	75	5	3
12	F	53	5	4.8	5	90	0	1
13	F	40	11	3.8	10	90	2	1
14	F	59	19	4.7	2	90	0	3
15	M	23	25	2.4	0	90	2	2
16	M	32	18	3.9	0	75	0	6
17	M	49	6	2.6	10	90	3	10

**Table 2:** Overview of the clinical and histopathological characteristics of patients without adenoma progression. *∗* indicates residual adenoma size as measured at the largest diameter; n.d.: could not be determined.

Patient	Sex	Age	Residual adenoma size, mm*∗*	Monitoring time, years	p21 expression	p27 expression	p53 expression	Ki-67 expression
1	F	67	26	9.8	0	25	0	1
2	F	47	11	9.1	n.d.	n.d.	n.d.	1
3	M	62	4	8.5	3	90	10	1
4	M	65	34	8.8	3	75	1	1
5	F	56	27	8.9	1	90	1	1
6	M	62	14	7.2	25	50	5	1
7	M	64	13	7.4	1	30	0	1
8	M	58	9	11.4	1	90	0	1
9	F	36	10	9.1	5	90	0	1
10	M	64	29	7.5	2	75	1	2
11	M	52	12	7.9	5	75	1	2
12	M	49	27	10.1	1	n.d.	0	1
13	M	53	3	10.4	0	75	0	2

## Data Availability

All anonymized clinical and histopathological data that have been analyzed are in Tables 1 and 2. So it is possible to perform second analysis. The samples of the patient which were analyzed are stored in Department of Pathology, University Hospital Ostrava (address: 17 Listopadu 1790, 708 52 Ostrava, Czech Republic.
